# An intervention to promote patient participation and self-management in long term conditions: development and feasibility testing

**DOI:** 10.1186/1472-6963-10-206

**Published:** 2010-07-14

**Authors:** Joanne Protheroe, Tom Blakeman, Peter Bower, Carolyn Chew-Graham, Anne Kennedy

**Affiliations:** 1 National Primary Care Research and Development Centre, University of Manchester, Oxford Road, Manchester, M13 9PL, UK

## Abstract

**Background:**

There is worldwide interest in managing the global burden of long-term conditions. Current health policy places emphasis on self-management and supporting patient participation as ways of improving patient outcomes and reducing costs. However, achieving genuine participation is difficult. This paper describes the development of an intervention designed to promote participation in the consultation and facilitate self-management in long-term conditions. In line with current guidance on the development of complex interventions, our aim was to develop and refine the initial intervention using qualitative methods, prior to more formal evaluation.

**Methods:**

We based the intervention on published evidence on effective ways of improving participation. The intervention was developed, piloted and evaluated using a range of qualitative methods. Firstly, focus groups with stakeholders (5 patients and 3 clinicians) were held to introduce the prototype and elucidate how it could be improved. Then individual 'think aloud' and qualitative interviews (n = 10) were used to explore how patients responded to and understood the form and provide further refinement.

**Results:**

The literature highlighted that effective methods of increasing participation include the use of *patient reported outcome measures *and *values clarification exercises*. The intervention (called PRISMS) integrated these processes, using a structured form which required patients to identify problems, rate their magnitude and identify their priority. PRISMS was well received by patients and professionals. In the individual qualitative interviews the main themes that emerged from the data related to (a) the content of the PRISMS (b) the process of completing PRISMS and how it could be operationalised in practice and (c) the outcomes of completing PRISMS for the patient. A number of different functions of PRISMS were identified by patients including its use as an aide-memoire, to provide a focus to consultations, to give permission to discuss certain issues, and to provide greater tailoring for the patient.

**Conclusions:**

There was evidence that patients found the PRISMS form acceptable and potentially useful. The challenge encountered by patients in completing PRISMS may encourage exploration of these issues within the consultation, complementing the more 'task focussed' aspects of consultations resulting from introduction of clinical guidelines and financial incentives. Further research is required to provide a rigorous assessment of the ability of tools like PRISMS to achieve genuine change in the process and outcome of consultations.

## Background

The global burden of disease is shifting to long-term conditions,[1] and there is worldwide interest in the development of models of service delivery to manage these changing needs[2]. UK Government policy places emphasis on self-management as a means of improving long-term conditions, and supporting patient participation in healthcare is seen as a key mechanism to improve self management[3,4]. Participation in health care has been defined as:

'an interaction, or series of interactions between a patient and the healthcare system or health care professional in which the patient is active in providing information to aid diagnosis and problem-solving, sharing his/her preferences and priorities for treatment or management, asking questions and/or contributing to the identification of management approaches that best meet his/her needs, preferences of priorities[5].

This acknowledges the patient as 'co-producer' of their health and integrates them as a key participant in the care process[6].

**Figure 1:**
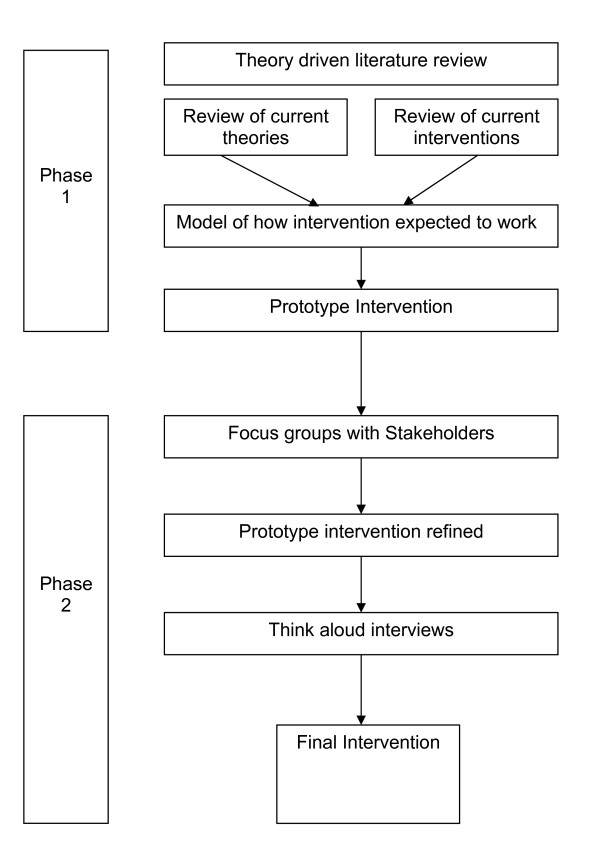
**Structure of the intervention development process**.

In this paper we describe the development of an intervention intended to promote participation in the consultation and facilitate self-management in long-term conditions. In line with current guidance on the development of interventions,[7] our aim was to develop an intervention that was informed by the existing literature, and refined using qualitative methods (see Figure 1). The development of the intervention described in the paper is set in the context of a larger study - the WISE approach (Whole Systems Informing Self-management Engagement) [8] designed to improve the way primary care services provide self care support for patients with long-term conditions, particularly for those who live in disadvantaged areas[9].

Previous work by the first author has questioned the assumption that interventions designed to enhance participation result in health improvements[10]. Some studies have reported that patients who actively participate in consultations with their health professionals have measurably better health outcomes than those who do not, both physiologically (blood pressure, blood sugar) and subjectively (evaluations of overall health status) [11-13].

A systematic review of intervention strategies designed to enhance patient participation found more positive effects of participation interventions in the outcomes of communication, provider diagnosis and process measures of the management of patient conditions. They found less effect on health status, patient satisfaction, self-efficacy and attitudes and behaviours such as adherence to treatment[5].

This review found that the most commonly used patient intervention to promote active participation was feedback to providers of *patient reported outcome measures *(PROMs). PROMS were reported to have had a positive impact on processes of care (i.e. communication between patient and health care provider, concordance), although there was less impact on patient satisfaction and health status.

Despite the positive evidence on PROMS, it has been noted in the literature that there is a lack of clarity on how and why PROMS might work, and their full potential in clinical practice is unclear because it is not made explicit how they would contribute to changing the nature of the relationship between patients and health professionals over time[14].

Another type of intervention reported to be effective at promoting patient participation was a *values clarification exercise *(VCE). VCEs are intended to help patients to first think about and then communicate the personal importance of different negative and positive features of 'options', in order to improve the match between what is personally most desirable and the option actually selected. Values clarification has been demonstrated to have a substantial effect on concordance, and has been closely linked to the use of decision aids[15].

There are a number of ways in which values clarification can be achieved. These include describing the features and likely outcomes of the options in sufficient detail to enable the patient to fully understand what is involved; giving examples of patient stories of how other patients' values led them to make certain choices; or explicitly measuring the patients' individual values for options. Some interventions encourage patients not only to clarify their values but to share them with important others such as family members and/or health professionals[16].

In line with the definition of participation used by Haywood et al [5] (see above) it could be hypothesised that the mechanism by which PROMS can improve participation is through active communication, "the patient is active in providing information to aid diagnosis and problem-solving" and the mechanism by which VCE can improve participation is by clarifying patient values, "sharing his/her preferences and priorities for treatment".

### Initial development of the intervention

The literature review highlighted the role of PROMS and VCE as mechanisms to improve patient participation, and the initial version of the intervention in this study was designed to contain components of both. However, unlike many 'PROMs', this intervention is not intended to be used as an outcome measure; rather it will be a mechanism to promote participation in the consultation, and by clarifying patient values, establish patient priorities for action in order to facilitate shared decisions about appropriate self care support.

The first version of the intervention combining PROMs and VCEs was called 'PRISMS' (Patient Report Informing Self-Management Support - see Figure 1). PRISMS is intended to encourage patients to consider what problems and issues are important to them, to support joint exploration of patients' problems and needs with their health professional and help with the establishment of agreed priorities. This aims to ensure that effective shared decision-making and the negotiation of an agreed plan of action can occur with their health care provider. Completing PRISMS requires three decisions - which problems are relevant to the patient, the magnitude of the problem, and their relative priority.

**Figure 2:**
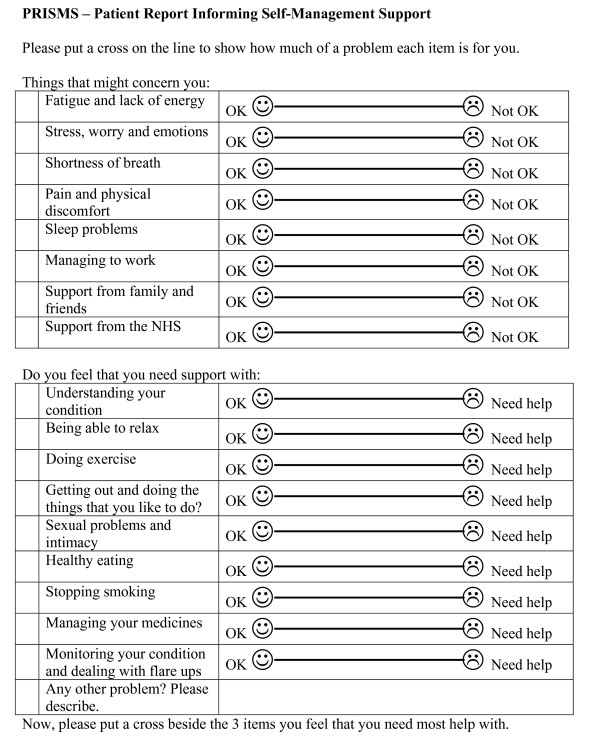
**Initial version of PRISMs**.

Having developed a first version of the intervention (see Figure 1), based on evidence from the literature, the aim of this study was to further develop and evaluate the intervention to enhance patient participation in their health care (see Figure 2). This was achieved using mixed qualitative methods of a) focus groups of stakeholders (patients and clinicians) and b) piloting the intervention using 'think aloud' and qualitative interview techniques.

## Methods

### Refining the intervention using focus groups

The focus groups with key stakeholders (patients and clinicians) were formed with a wider remit to explore the components and acceptability of the WISE approach. The initial version of the PRISMS intervention was refined through these focus groups. The aim of the PRISMS questions in the focus groups was to assess patient and professional perspectives on PRISMS, and to explore the barriers and facilitators to adoption in everyday practice.

#### Sample

The patient sample was taken from our key target group, people with long-term conditions living in socio-economically disadvantaged communities. We targeted the condition Chronic Obstructive Pulmonary Disease because this disproportionately affects people in lower socio-economic groups. We drew the sample from people attending a pulmonary rehabilitation class based in an inner city Primary Care Trust with high levels of social deprivation. All the patients attending the pulmonary rehabilitation class on the particular day that the researcher (AK) visited were invited to take part in the study. Health professionals were sampled from practice nurses in the same PCT; all the practice nurses with an interest in COPD were invited to take part in the study.

#### Procedure

During the focus groups, patients were asked to complete the prototype PRISMS and the following prompts were used to encourage discussion after they had completed it: is there anything missing from the PRISMS form?; are the things you consider you need most help with the same things that your doctor or nurse thinks you need help with?; do you ever have disagreements with your doctor or nurse about your COPD management or is there anything you think they don't help you with? The researcher observed the patients during this process and noted any problems with completing the form and queries from individuals, in order to identify any immediate barriers.

Professionals were shown the PRISMS form, and the following prompts were used to encourage discussion: is there anything missing from the PRISMS form?; do you think patients will consider they need most help with the same things that you do?; do patients ever disagree with you about their condition management?; how do you see PRISMS being used and when?

#### Analysis

The focus groups were recorded and transcribed. Data from the transcripts which referred to the use of the PRISMS forms was selected and together with field notes about how the patient group filled in the forms was used to refine the prototype intervention prior to its inclusion in the 'think aloud' and qualitative interviews described below.

### Piloting the intervention using 'think aloud' and qualitative interview techniques

The second study was designed to provide a more detailed exploration of the way patients understood and responded to PRISMS when thinking about their long-term conditions, in order to better understand how the intervention might encourage new ways of thinking and thus meet the aims of improving participation, communication, decision making and self management.

One method chosen to achieve this was 'think aloud'. Unlike traditional qualitative interviewing, 'think aloud' involves less of a dialogue between interviewer and respondent. Rather, the focus is on respondents verbalising their ongoing thoughts during a task, as a way of accessing their decision making processes[17,18]. The function is to examine the content and order of information processing during the task. The 'think aloud' interviews were complemented by more conventional semi-structured interviews after the 'think aloud' process was complete.

#### Sample

Patients were recruited from practices based in the same PCT where the focus groups were held. Recruitment was in the context of an exploratory study of a training programme for clinicians for the management of long-term conditions (part of the study on the WISE approach). Patients in the WISE study were either on the chronic disease registers for chronic obstructive pulmonary disease or diabetes, or were diagnosed with irritable bowel syndrome. Potential participants were identified by practice staff at the reception desk and asked if they were willing to find out about a research study. Interested potential participants were contacted by a researcher on the telephone and given details about the interview study and invited to participate.

#### Procedure

Patients consenting to be interviewed were invited to attend for a face-to-face structured interview at their own general practice. All the patient participants were interviewed by the first author (JP). Data were collected on participant demographics, clinical conditions and management. They were then shown the PRISMS and asked to complete it, while being encouraged to talk aloud about their thinking and decision making as they tried to fill it in. Verbal prompts were used such as 'What are you thinking?' 'Tell me why you put that?'. These prompts referred only to the current exercise of completing the questionnaire.

After completing the PRISMS and the 'think aloud' task, patients were then interviewed using the following prompts outlined in Box 1. This part of the interview allowed patients to explore potential anticipated future use of the instrument.

Box 1: Interview Prompts

• Is it understandable?

• Are there any difficulties filling it in?

• Would any difficulties be overcome by filling it in with a professional (e.g. literacy problems)?

• Would they prefer to complete PRISMS pre-appointment or during an appointment with a professional?

• Does it cover what concerns the patient most about their condition?

• Does the priority setting exercise make sense?

• Do patients feel it would alter the way their usual review appointments proceed?

In the early stages of the study, the researchers became aware that patients' responses to PRISMS seemed to be influenced by social desirability, as it was clear to most patients that the research team were involved in the development of PRISMS. To reduce this influence, an alternative publicly available PROM was used in addition to the prototype PRISMS to allow comparisons to be drawn. The participants were not made aware which of the two interventions was of particular interest to the research team.

#### Analysis

Interviews were continued until category saturation was complete. All the interviews were audiotaped and transcribed verbatim. Data were analysed using a framework analysis by JP and AK [19]. An initial coding framework was developed with reference to the transcripts, the original study protocol, the research question and the literature underpinning the intervention. The transcripts were checked against the framework to ensure that there were no omissions. Codes in each interview were examined across individual transcripts and the entire data set and allocated to the framework. The categories were refined and broader concepts emerged from the data linking codes across the interviews. Data were interpreted and analysed within the framework leading to the emergence of several themes related to the intervention.

Ethical approval for the study was obtained from Oldham LREC (ref 07/H1011/96).

## Results

The results of both the focus groups and the 'think aloud' qualitative interviews are presented in this section. For ease of presentation the quotes supporting the results are displayed in boxes. When the quotes represent more than a single speaker, the researcher is identified as 'A' and the participant is identified as 'B'. All the quotes are referenced by a unique, anonymous identification number.

### Refining the prototype intervention using focus groups

In the patient focus group five out of the seven participants at the pulmonary rehabilitation class took part in the focus group; two patients had prior engagements. Demographic data were not formally gathered, although all the participants were female and of middle-age or older. In the health care professional focus groups, three practice nurses with a special interest in COPD consented to take part.

The focus groups provided useful data on the general reaction to PRISMS among stakeholders. In particular, observations showed that patients encountered the following immediate barriers to use of the early version of the form:

1. Vision and health literacy issues - some patients had difficulty reading the form, and others found the language difficult to understand.

2. Patients reported difficulty in determining whether the issues listed on PRISMS really were a concern to them or if it was satisfactory because they just 'coped' and did not expect additional help.

3. Patients reported that it was hard to separate out problems they had with COPD from problems with their other conditions

However, the concept behind PRISMS - that of bringing the patient's needs into the consultation - did seem important and novel to the participants.

The practice nurses readily picked up on how to use the PRISMS tool and incorporate it into their everyday practice:

1. Nurses reported a 'tick-box' mentality in current clinical work to fulfill the requirements of the Quality and Outcomes Framework (QOF). This activity was viewed as being peripheral to patient's quality of life and incompatible with the patient agenda. The PRISMS was seen as an opportunity to re-focus on the patients agenda.

2. Overall the content of the PRISMS form was seen as appropriate.

3. PRISMS may make it easier to get better focus on management of co-morbidities

4. There was a view that they and patients will find it hard to interpret the question on support from the NHS

In the light of these findings, the PRISMS form was shortened, simplified and the font size was increased.

### Piloting the intervention using 'think aloud' and qualitative interview techniques

Ten patients from two practices consented to participate in the 'think aloud' and qualitative interviews. Most interviews lasted between 30 and 45 minutes. The socio-demographic characteristics of the study participants are summarised in Additional File Table 1. The main themes that emerged from the data related to (a) the content of the PRISMS (b) the process of completing PRISMS and how it could be operationalised in practice and (c) the outcomes of completing PRISMS for the patient.

**Table 1:** Demographics of participants in "Think aloud" study

Pt id	Sex	Condition	Age	Ethnicity	Employed	Job	Partners job	Home	Highest qualification
W001	F	IBS recent diabetes (2 weeks)	65	White	No	retired	Retired teacher	owned	GCE
W003	F	Asthma (10 years)	83	White	No	Used to work in payroll	None	Owned	No formal- apprenticed in accounts
W004	F	IBS (20 years)	39	White	Yes	Pharmacist/lecturer	Pharmacist/Director	Owned	PhD
S001	F	Diabetes (2.5 years)	67	White	No	Retired VDU operator	Retired engineer	Owned	None
S002	M	Diabetes	74	White	No	Retired window cleaner	No	Owned	None
S004	F	IBS	80	White	No	Retired		Owned	None
S005	F	IBS	76	Black-Caribbean	No			Owned	GCSE English
S006	M	Diabetes	79	White	No	Retired railway worker	None	Rented	None
S007	M	COPD	62	White	No			Owned	HND - chemical engineering
S008	F	COPD	70	White	No	Retired - taught stained glass at night school	Health and safety	Owned	None - craft

#### (a) The content of PRISMS

**Figure 3:**
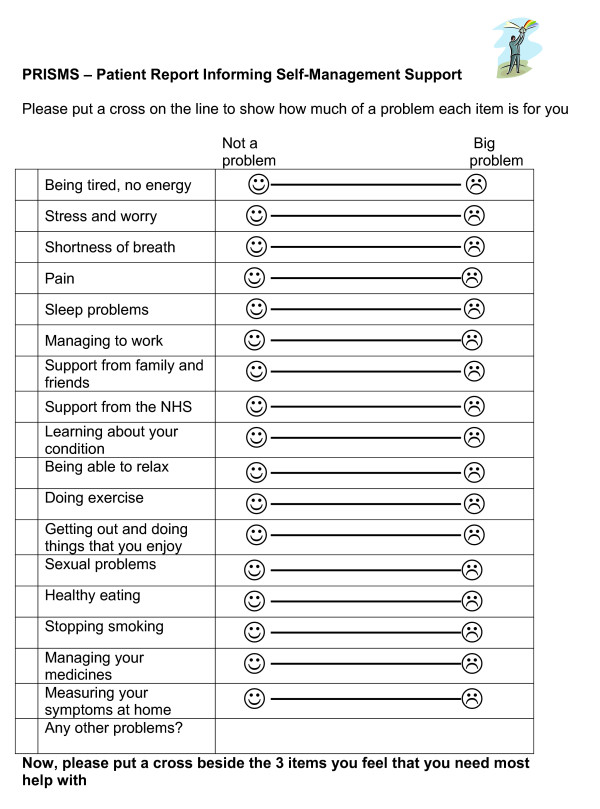
**Final Version of PRISMs**.

The content of the PRISMS form used in the interviews is represented in Figure 1, however, as a result of the focus groups, the wording was simplified and the font size increased (Figure 3).

It was felt that the topics in PRISMS were comprehensive and covered the majority of issues in long-term conditions. As noted earlier, the study included a comparison between the PRISMS form and another patient-oriented PROM. A major difference was that PRISMS included a list of issues for patients to consider, whereas the comparison used an open ended method of simply asking patients if they had any problems or concerns. The prompts were preferred by the majority of patients. (See Box 2)

Patients were comfortable with the idea of quantifying how much of a problem something was using a line with a sad face or a smiley face. Only one patient in the group preferred a numeric format.

Box 2: The content of PRISMS: Quotes:

A OK. So do you think it covers a wide enough range of... Of problems that people might have with a... you know... with managing their ... medical conditions and...?

B yes because you've got general things like healthy eating, stopping smoking, and... managing your medicines. Those are general things, aren't they, which could apply to all sorts of things

A uhm

B erm but yeah, I would say that erm... that you've more or less covered everything there.

ID W001

B ***[reading off comparative form] ***'name two symptoms that bother you most write them on the lines....concerning how bad each symptom is over'.... What symptoms are we referring to though?

A Well I think you have to choose your own symptoms.

B Because at the moment, I mean ... [sighs] ***[reading again]***.... As good as it could be... as bad as it could be

A I think... imagine you were filling this out before your next review appointment really.

B Right. So ... I don't feel any different between now and when I had it done in... February.

ID S002

#### (b) The process of completing PRISMS and operationalising in practice

Completing PRISMS requires three decisions - which problems are relevant to the patient, the magnitude of the problem, and the relative priority among problems. In relation to the first decision, the content of PRISMS was felt to cover all the main problems of relevance to patients with long-term conditions. However, in deciding which problems to highlight, several issues impacted on their decision making. First, long-term conditions were characterised by their *variability*, which meant that a single rating could only ever be a crude approximation to changes over time. There were problems with making judgements about how their experience related to the categories on the PRISMS. Patients also reported some difficulties distinguishing problems associated with their long-term conditions, and those related to advancing age. Adding to that was the difficulty of judging the relevance of a problem which might be controllable with medication. Finally, patients distinguished between other life problems, and problems requiring a health care intervention. (See Box 3)

Box 3: The process of completing PRISMS - Decisions

Quotes:

B Er I did once I, once I'd sort of sorted that out with... erm... ***[reads] ***put a cross on the line to show how much of a problem each item is for you. Its quite self-explanatory that any way.........

Right erm... put a cross on the line to show how much of a problem each item is for you. Ah ...right well being tired er... erm... I don't lack energy but I do feel tired especially in the evenings erm... so I'm putting a... I would say probably about half way.

ID W001

B Stress and worry. Hum. Well that's very time dependent I suppose because if I'd filled this in two weeks ago that would have been probably on the right hand side of the sad face [laughs] but at the moment its, its erm... fairly, I'll put it in the middle though 'cos there are some on-going things.

ID W004

B Erm... I'm figuring out where they overlap or where they don't. Like the erm... well certainly in the first table like the stress and worry and the pain and stuff like that, you know, some of these things get very strongly linked in your mind erm... but they're separated there so I'm not quite sure how you could ...

ID W004

B It is, it's awkward to know what is to do with the Diabetes and what is to do with my age.

ID S001

B 'Stress and worry' not a problem, 'Shortness of breath' ... only if I run, if I run. Not a problem. 'Pain' well the only pain I get is in a morning down the back of me thighs, when I get out of bed and then I shuffle off and I have me medicine and then it goes. Its no... not a problem.

ID S006

B ...the sort of the going, getting out and doing exercise and things that is, that is you know, individual isn't it, its down to the person, the doctor can't really help you with that. Short of giving you a walking stick or... ...What about stress and worry though? I mean a doctor can't do much about that can he?

ID W003

B "do you need help with getting out and doing things that you enjoy." Actually that, that was odd for me to see on that list, and actually I don't know whether I might have expected to see it ..maybe instead of that managing to work, that I did. That... 'cos it didn't feel like something that I'd necessarily expect my doctor or nurse to help me with.

ID W004

**Figure 4:**
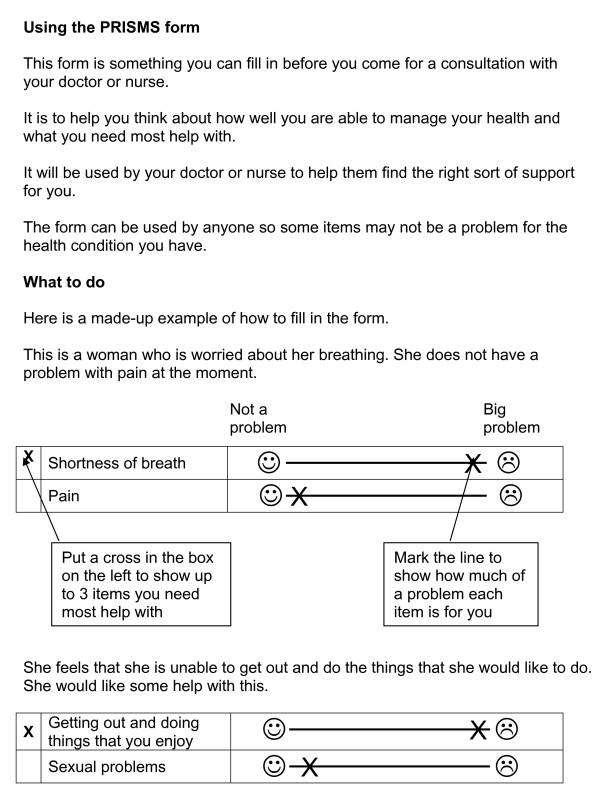
**Patient Instructions with PRISMs**.

The instructions asked patients to consider all the proposed areas for action, and mark along the line (see Figure 4) how much of a problem each area was. They then were asked to mark out the three areas that they would like to address first. It was noted that the three items chosen were not always the three items that the patient had marked as the greatest problems.

Patients prioritised those problems that they thought would be helpful to discuss at their next review appointment. Other patients discounted those problems that they felt were unrelated to their health, or that they already had plans for dealing with. Patients found that using the form and then reflecting on their answers by attempting to prioritise three problems helped them to identify where their main issues were and reflect on health issues as they related to the rest of their life, rather than in a condition-specific way (as is the norm in a consultation). See box 4.

Box 4: The process of completing PRISMS - Prioritising

Quotes:

A and shortness of breath is on... you marked it as a bigger problem, but is it not something that you'd want to... you feel you need any help with or.

B Not really, because I don't, I, I don't, I don't know how... I think... I don't know how anybody else could help me with that.

A Right. So maybe that that would be helped more by learning about your condition

B Yes, yes

A that you did tick.

B Yes, yes.

ID W003

A I noticed just that, so that we understand how its been filled. You... the ones that you've ticked aren't necessarily the ones that are the biggest problem. Is it... have you... was there a reason for that?

B erm... I suppose the reason being that erm... while pain isn't a problem at this moment in time, 'cos I can manage really, I can manage it, I can alleviate it.

A Yes

B there are times when I can't.

ID W003

B OK being tired, no energy [laughs] yes .......also you start to wonder about... you know I'm a mum of two small boys so... you that is actually a huge problem but its probably nothing to do with my IBS at all.

ID W004

B its uhm... there's only really pain and sleep problems that there's any ...

A Well just put two crosses then I think

B Here?

A Yes.

B I don't seem too bad do I? [laughs]

ID S001

B Well, my first thought was ...it makes you sit back and wonder whether you are... *well *actually. ***[in comparison to others]***

ID S007

Although the actual process of completion of the PRISMS was unproblematic, discussions following the 'think aloud' highlighted that the process of prioritizing problems in a formal way was an innovation that, although not unwelcome, would require a significant culture change in primary care, particularly from the patients' perspective, if it was to be routinely included in reviews of long-term conditions.

The majority of patients felt that they would prefer to complete a form like PRISMS at home in advance of their review appointment, although a minority felt comfortable do so either at home or during an appointment. They also felt that it would be useful as a regular exercise, either annually or on a six-month basis, depending on when their chronic disease review appointments were. See box 5.

Box 5: The process of completing PRISMS - Operationalising in practice

Quotes:

B Fill it in. Erm...I .... I think its, yeah definitely one of the clearer things that I've, I've seen, like this. I think its quite simple. I think that like me, if you're, if you're confronted with something that you don't see as being part of your problem, then again, its kind of a culture change, you know sort of like .......

I suppose the big thing about it is it just seems like a real change in, you know, given something proactively before you go for a consultation with the doctor or nurses.

A uhm

B you know, a bit of a change in culture really. You expect to just come in, do what you do in the consultation and walk out again. Erm.. I think it's a very good thing. Erm but I suppose if I was hit with it without erm... a bit more of the information that perhaps is in there about what the, what the aim of this is more broadly, you know about, you know

ID W004

B ... filling it in with the Doctor you could discuss it while you're filling it in. But it's the same if you fill it in at home

A uhm

B even its there for the Doctor

A So you wouldn't mind either way

B no, no.

A Right. And if it was sent to you with your Review appointment

B you'd fill it in first before you ...

ID S001

B ...filled it in and bought back in. yes its no problem that.

A Or filled out in the appointment with the doctor or the nurse?

B no... I think sent out with the appointment time I think is better, yes, yes.

A Any particular reasons for that?

B erm... probably 'cos I've got plenty of time on the side...

ID S007

B Erm.. yes perhaps once every six months. Erm... I suppose after a time those, those problems wouldn't be the same would they again, six months later?

A No. So you might be ticking a different three.

B Yes.

IDW003

#### (c) The outcomes of completing PRISMS

Patients felt that using the PRISMS would have a number of potential benefits. PRISMS could act as an aide-memoire for key issues that they wanted to discuss. PRISMS could also act as a focus for review appointments, providing a structure for the consultation. This might make the consultation more efficient, but reducing the need for routine questions to focus on patients' particular needs. See Box 6.

PRISMS might also have a more subtle function. Patients had the perception that primary care professionals were always busy, and the presence of the PRISMS form could provide permission to talk about issues that might otherwise not be raised. PRISMS could also make the consultation more patient-centred and focussed on the needs of the Individual patient.

Box 6: The outcomes of completing PRISMS

Quotes:

A tell me a bit why you think that might make a difference.

B Well when you got in the Doctor's sometimes you have about three things to mention and you come out many a time thinking gosh I should have said about that

A uhm

B you know. You don't... you don't mention it.

A right. so this would provide...

B Yes that would er... provide like the opening to remind you to...do it.

ID S001

A do you think that it would affect the way your appointment went?

B ... yes I would think so, I mean there'd be definite things to talk about then wouldn't there?

A uhm

B not just a case of ... how are you today and I say alright. [both laugh]

ID S004

B ...that is easier on both sides. ***[doctor and patient] ***The doctor just has to look where the ticks are, or the crosses are, and see what your problems are.

S008

B yes... if I did that on a once a year appointment and took it to H the diabetic nurse and she looked at that, she said "oh right well everything's OK" or you know...

A uhm

B whereas she wouldn't need to ask all the different questions. She'd still have to do the other side of it **[*refers to blood and urine testing for diabetes]***

S002

B and another thing, I mean you always feel, they are in a hurry aren't they, these days, he's very, very nice I think I've half mentioned that, you know, of course I don't.

A uhm.

B so...so that's my fault!

A but having something with you that, that you've written down

B that's right, yes.

A might help with that

ID S004

B yes certainly, and it, its, its in relationship to me, isn't it this?

ID W003

A OK. That's fine. And do you think that it makes sense to you to pick three out of all of them... to be sending back?

B Yes. Erm... 'cos you're getting the most important, most important ones to the person

A uhm

B outlined aren't you?

ID W003

## Discussion

### Summary

Previous work by the authors, reviewing the literature, suggested that an effective intervention for increasing participation might include both PROMs and VCE. We designed a prototype intervention (PRISMS), which included aspects of both these interventions, which underwent preliminary feasibility testing with patients and professionals using qualitative methods.

PRISMS was well received, and patients and professionals could see value in having it introduced into their review appointments for chronic disease. As with any tool, there were individual preferences for content and format, and patients provided useful feedback for redrafting the instrument to assist in its completion.

The think aloud interviews highlighted the complexity of the decisions behind the simple responses on the form, and the findings echo previous work on patients completing ostensibly simple satisfaction questionnaires and outcome scales[20,21]. For example, in an analysis of respondent's decision making in completing the SF36, Mallinson highlighted the complex comparative judgements underlying assessments of general health, or the changes over time related to increasing burden of illness that can influence the way in which assessments are made. Of course, such variability is more of an issue for the SF-36, which is designed as a *standardised *assessment of outcome. For PRISMS, it would be necessary for health professionals to understand something of the process by which patients come to their decisions. However, the difficulty patients reported in identifying 'problems', rating their magnitude and identifying their priority may encourage patients to reflect on these complex issues. If delivered in parallel with training for professionals, PRISMS might act as a platform to allow exploration of these issues, rather than the 'task focussed' consultations that have resulted in part from the Quality and Outcomes Framework that has had such a profound impact on consultations in the United Kingdom[22-24]. The intention of the PRISMS intervention is that it would be used as a starting point for discussion of patient priorities in a consultation, and not as an outcome. As well as allowing the patient to express needs and concerns, PRISMS can also be used by clinicians as a non-threatening way to challenge patients' perceptions of their behaviour and need for support (for example the problems of smoking and diet). We acknowledge that there would be a danger of this also becoming a so called 'tick-box' exercise, for this to be avoided it would be necessary to link the introduction of the PRISMS form with professional training.

Patients also identified a number of different functions of PRISMS (i.e. as an aide-memoire, to focus consultations, to give permission to discuss certain issues, and to provide greater tailoring for the patient). An important finding was that patients often did not prioritise their main problem, in the sense that it was not seen as being appropriate for discussion with the professional. This may have been related to general low expectations of what the NHS can do in terms of support for many issues.

The literature identified several issues that need to be addressed in order to maximise the effect of PROMS used in an intervention. These include incorporating the patients' perspective, feeding the data back through the decision making process and assessing if the patients and providers value the information provided. The evidence outlined in this paper has begun to address those issues, however, more research and development is required to provide a more comprehensive assessment. In relation to including the patient's perspective, the interview data suggested that the PRISMS form included areas that were important to the patients. There is free space on the form for patients to note down any additional areas that they would like to address in their review appointment. The form is designed to be used in a repeated manner at future review appointments, where patients will have the opportunity to express different priorities for action. This was highlighted in the interview data as a useful exercise. Further research will be required to test how much patient priorities change, and whether PRISMS allows them to express those changes in a useful manner.

This study provided only preliminary evidence of acceptability to professionals. A key research question is whether the PRISMS form provides sufficient detail for professionals immediately, or whether it functions as a stimulus to further discussion about priorities. A longer-term issue is whether the PRISMS can be made available to, and used by clinicians and service providers outside primary care. There is evidence from the patient interviews that patients value using the PRISMS as a basis for a patient centred, priority focused review appointment. However, a critical question for the future is whether those immediate reactions can be translated into longer-term changes to the process of care and patient outcomes.

### Study limitations

The sample size used in the individual interview study was relatively small, because the focus was on the collection of rich data involving both think aloud and interview methods. This may have the disadvantage that we have not been able to access some groups of relevance, such as ethnic minorities, or specific conditions, such as depression, which may raise other issues. As noted earlier, there was a perception that some responses to PRISMS may have reflected socially desirable responses, with patients aware that the team was involved in the development of the PRISMS, although this effect was mitigated by the introduction of the comparator tool after the initial interviews, to encourage a more critical approach. Most participants were over the age of 60 years; however, this reflects the population of patients with long-term conditions. Younger patients might have different expectations about the outcomes of consultations and be more proactive in seeking support without the need for the legitimization provided by PRISMS. The initial focus group of stakeholders was also small and only included nurses, it is possible that other health care providers may have differing views on the use of PRISMS, this will require further evaluation.

### Policy and practice implications

PRISMS is designed to encourage consideration of problems and priorities from a patient perspective and thereby encourage participation in making decisions about their management. The introduction of PRISMS is likely to be complex, but there are lessons that could be learned from related interventions. For example, primary care practitioners are now incentivised to use depression screening measures in patients with selected long-term conditions. Although there is some professional resistance, patients report that they are more positive about their use, seeing them as 'efficient and structured supplement to medical judgment and as evidence that general practitioners were taking their problems seriously through a full assessment'[25].

PROMS, VCE and PRISMS are all interventions that are well placed within current policy around the management of long-term conditions in the United Kingdom. As well as the focus on participation and self management, PRISMS provides a potential model for *care planning*, to which the UK Department of Health has made a policy commitment, such that 'everyone with a long-term condition has a personalised care plan'[26]. Care plans are designed to be agreed by patient and professional to organise packages of care that are personal to the patient, and regularly reviewed.

Care plans form an important part of the Chronic Care Model,[27], and of the 'care programme approach' in mental health, and have been used in disorders such as asthma [28]. Evaluations of care planning suggest that it can be an effective component of care,[29-31] and there is emerging evidence concerning the optimal ingredients[32]. However, there is no consensus as to the best way to implement care planning across different long-term conditions. Experience abroad has highlighted variation in content of care plans,[33] and qualitative studies have also suggested some professional and patient ambivalence,[34] and limited impacts on collaborative self care and co-ordination between professionals[35,36].

One of the potential limitations of care plans up to now is that they have been focused on professional issues and concerns, rather than those raised by the patient. The focus on issues of priority in PRISMS may serve to readdress this imbalance. Recent commentators have highlighted how patients with long-term conditions have to deal with multiple management regimes and a significant burden of *treatment*[37]. This is a particular problem in patients with co-morbidities[38]. However, many patients are not used to considering interactions between different conditions and their management,[39] and many want help and support in prioritizing the competing demands from multiple conditions[40]. Identifying priorities and helping patients deal with tensions between different management options has been identified as a core issue for effective clinical practice in the context of long-term conditions[37,41]. Tools like PRISMS may function as a platform to explore priorities.

### Future research

As noted earlier, the impetus for the development of PRISMS was evidence from a systematic review of interventions by Haywood et al showing that PROMS and VCE were effective in improving patient participation[5]. However, the evidence for that effectiveness is inconsistent, and more related to the process of care than outcomes. A recent review of interventions to encourage question asking (through both written prompts and coaching) also reported modest impacts on the process of care which did not generalize to other outcomes[42]. Although this qualitative study found that patients and professionals were broadly supportive, and this study focused on patient accounts of how the PRISMS form *might *be used, translating such attitudes into demonstrable changes in clinical behaviour and patient outcomes is a significant challenge.

However, it should be noted that PRISMS is designed to be used as part of a multifaceted intervention at different levels which are interlinked to maximize impact. At the patient level, PRISMS is designed to improve patient participation in care. This will be enhanced by professional training in patient-centred consultations and self management support, which in turn will be augmented by interventions at the level of the health system, which are designed to provide better access to self management support within primary care. A randomised trial is ongoing to test whether this combination achieves the planned increases in participation and self management, and whether those gains are translated into improved health outcomes and reduced costs.

## Conclusions

This study provides evidence that patients found the PRISMS form acceptable and potentially useful. The challenges encountered by patients in completing PRISMS may encourage further exploration of these issues within the consultation, complementing the more 'task focussed' aspects of consultations resulting from introduction of clinical guidelines and financial incentives. Further research is required to provide a rigorous assessment of the ability of tools like PRISMS to achieve genuine change in the process and outcome of consultations.

## Competing interests

The authors declare that they have no competing interests.

## Authors' contributions

All authors read and approved the final manuscript. All authors commented on the paper and contributed to the final draft. JP conducted the literature review and JP and AK drafted the protocol intervention, refined by TB and CCG. JP, TB, PB, CCG and AK were all involved in the original design and writing of the protocol, and contributed to the critical reflection of data and feedback which led to redesign of the intervention. JP and AK were responsible for the data collection.

## Pre-publication history

The pre-publication history for this paper can be accessed here:

http://www.biomedcentral.com/1472-6963/10/206/prepub

## References

[B1] Murray CJ Lopez AD The global burden of disease:a comprehensive assessment of mortality and disability from disease, injuries and risk factors in 1990 1996 Boston: Havard School of Public Health on behalf of the World Bank

[B2] Epping-Jordan J Pruitt S Bengoa R Wagner E Improving the quality of health care for chronic conditions Qual Saf Health Care 2004 13 299 305 10.1136/qshc.2004.010744 15289634 PMC1743863

[B3] Department of Health Supporting people with long term conditions: An NHS and Social Care Model to support local innovation and integration 2005 London: Department of Health

[B4] Department of Health The NHS Improvement Plan: Putting People at the Heart of Public Services. London 2004

[B5] Haywood K Marshall S Fitzpatrick R Patient participation in the consultation process: a structured review of intervention strategies Patient Educ Couns 2006 63 12 23 10.1016/j.pec.2005.10.005 16406464

[B6] Hibbard JH Engaging health care consumers to improve the quality of care Med Care 2003 41 I61 I70 10.1097/00005650-200301001-00007 12544817

[B7] Medical Research Council A framework for development and evaluation of RCTs for complex interventions to improve health 2000

[B8] Kennedy A Rogers A Bower P Support for self care for patients with chronic disease BMJ 2007 335 968 970 10.1136/bmj.39372.540903.94 17991978 PMC2071971

[B9] Kennedy A Chew-Graham C Blakeman T Bowen A Gardner C Protheroe J Rogers A Gask L Delivering the WISE (Whole Systems Informing Self-Management Engagement) training package in primary care: learning from formative evaluation Implement Sci 2010 5 7 10.1186/1748-5908-5-7 20181050 PMC2841580

[B10] Wetzels R Harmsen M Van WC Grol R Wensing M Interventions for improving older patients' involvement in primary care episodes Cochrane Database Syst Rev 2007 CD004273 17253501 10.1002/14651858.CD004273.pub2 PMC7197439

[B11] Kaplan SH Greenfield S Ware JE Jr Assessing the effects of physician-patient interactions on the outcomes of chronic disease Med Care 1989 27 S110 S127 10.1097/00005650-198903001-00010 2646486

[B12] Greenfield S Kaplan S Ware JE Jr Expanding patient involvement in care. Effects on patient outcomes Ann Intern Med 1985 102 520 528 3977198 10.7326/0003-4819-102-4-520

[B13] Brown R Butow PN Boyer MJ Tattersall MH Promoting patient participation in the cancer consultation: evaluation of a prompt sheet and coaching in question-asking Br J Cancer 1999 80 242 248 10.1038/sj.bjc.6690346 10390003 PMC2363012

[B14] Marshall S Haywood K Fitzpatrick R Impact of patient-reported outcome measures on routine practice: a structured review J Eval Clin Pract 2006 12 559 568 10.1111/j.1365-2753.2006.00650.x 16987118

[B15] Charles C Gafni A Whelan T O'Brien MA Treatment decision aids: conceptual issues and future directions Health Expectations 2005 8 114 125 10.1111/j.1369-7625.2005.00325.x 15860052 PMC5060284

[B16] IPDAS2005 Criteria for development and evaluation of patient decision aids 2005

[B17] Ericsson K Simon H Protocol analysis: verbal reports as data 1984 Cambridge: MIT Press

[B18] Boren M Ramey J Thinking aloud: reconciling theory and practice IEEE Transactions on Professional Communication 2005 43 261 278 10.1109/47.867942

[B19] Ritchie J Spencer L Bryman A, Burgess RG Qualitative data analysis for applied policy research Analysing qualitative data 1994 London: Routeledge

[B20] Williams B Coyle J Healy D The meaning of patient satisfaction: an explanation of high reported levels Soc Sci Med 1998 47 1351 1359 10.1016/S0277-9536(98)00213-5 9783878

[B21] Mallinson S Listening to respondents: a qualitative assessment of the Short-Form 36 Health Status Questionnaire Soc Sci Med 2002 54 11 21 10.1016/S0277-9536(01)00003-X 11820675

[B22] Charles-Jones H Latimer J May C Transforming general practice: the redistribution of medical work in primary care Sociol Health Illn 2003 25 71 92 10.1111/1467-9566.t01-1-00325 14498945

[B23] Campbell S McDonald R Lester H The experience of pay for performance in English family practice: a qualitative study Ann Fam Med 2008 6 228 234 10.1370/afm.844 18474885 PMC2384990

[B24] Checkland K Harrison S McDonald R Grant S Campbell S Guthrie B Biomedicine, holism and general medical practice: responses to the 2004 General Practitioner contract Sociology of Health and Illness 2008 30 788 803 10.1111/j.1467-9566.2008.01081.x 18444956

[B25] Dowrick C Leydon G McBride A Howe A Burgess H Clarke P Maisey S Kendrick T Patients' and doctors' views on depression severity questionnaires incentivised in UK quality and outcomes framework: qualitative study BMJ 2009 338 b663 10.1136/bmj.b663 19299474

[B26] Department of Health High quality care for all: NHS Next Stage Review final report. London 2008

[B27] Von Korff M Glasgow RE Sharpe M Organising care for chronic illness BMJ 2002 325 92 94 10.1136/bmj.325.7355.92 12114242 PMC1123637

[B28] Partridge MR Written asthma action plans Thorax 2004 59 87 88 10.1136/thx.2003.016451 14760139 PMC1746930

[B29] Gibson P Powell H Written action plans for asthma: an evidence-based review of the key components Thorax 2009 59 94 99 10.1136/thorax.2003.011858 PMC1746945 14760143

[B30] Zwar NA Hermiz O Comino EJ Shortus T Burns J Harris M Do multidisciplinary care plans result in better care for patients with type 2 diabetes? Aust Fam Physician 2007 36 85 89 17252093

[B31] Zwar N Hasan I Hermiz O Vagholkar S Comino E Harris M Multidisciplinary care plans and diabetes--benefits for patients with poor glycaemic control Aust Fam Physician 2008 37 960 962 19037474

[B32] Diabetes UK and the Department of Health Care Planning in Diabetes 2006 http://www.dh.gov.uk/en/index.htm

[B33] Vagholkar S Hermiz O Zwar NA Shortus T Comino EJ Harris M Multidisciplinary care plans for diabetic patients: what do they contain? Aust Fam Physician 2007 36 279 282 17392947

[B34] Jones A Pill R Adams S Qualitative study of views of health professionals and patients on guided self management plans for asthma BMJ 2000 321 1507 1510 10.1136/bmj.321.7275.1507 11118179 PMC27554

[B35] Blakeman T Harris M Comino E Zwar N Evaluating general practitioners' views about the implementation of the Enhanced Primary Care Medicare items Medical Journal of Australia 2001 175 95 98 11556428 10.5694/j.1326-5377.2001.tb143539.x

[B36] Shortus TD McKenzie SH Kemp LA Proudfoot JG Harris MF Multidisciplinary care plans for diabetes: how are they used? Med J Aust 2007 187 78 81 17635087 10.5694/j.1326-5377.2007.tb01144.x

[B37] May C Montori VM Mair FS We need minimally disruptive medicine BMJ 2009 339 b2803 10.1136/bmj.b2803 19671932

[B38] Bayliss E Steiner J Fernald D Crane L Main D Descriptions of barriers to self-care by persons with comorbid chronic diseases Ann Fam Med 2003 1 15 21 10.1370/afm.4 15043175 PMC1466563

[B39] Fried T McGraw S Agostini J Tinetti M Views of older persons with multiple morbidities on competing outcomes and clinical decision making Journal of the American Geriartrics Society 2008 56 1839 1844 10.1111/j.1532-5415.2008.01923.x PMC2596278 18771453

[B40] Bayliss E Edwards A Steiner J Main D Processes of care desired by elderly patients with multimorbidities Fam Pract 2008 25 287 293 10.1093/fampra/cmn040 18628243 PMC2504745

[B41] Bayliss E Bosworth H Noel P Wolff J Damush T McIver L Supporting self-management for patients with complex medical needs: recommendations of a working group Chronic Illness 2009 3 167 175 10.1177/1742395307081501 18083671

[B42] Kinnersley P Edwards A Hood K Ryan R Prout H Cadbury N MacBeth F Butow P Butler C Interventions before consultations to help patients address their information needs by encouraging question asking: systematic review BMJ 2008 337 a485 10.1136/bmj.a485 18632672 PMC2500196

